# Mesenteric Lymphatic Malformation Complicated by Torsion: A Case Report

**DOI:** 10.7759/cureus.92564

**Published:** 2025-09-17

**Authors:** Brayan Muñoz-Caicedo, Diego Ocampo, Daniel Mejía, Vanessa García Gómez

**Affiliations:** 1 Department of Radiology, Universidad de Antioquia, Medellín, COL; 2 Department of Radiology, Hospital Pablo Tobón Uribe, Medellín, COL

**Keywords:** abdominal pain, intra-abdominal cystic lymphangiomas, mesenteric cystic lymphangioma, mesenteric lymphangioma, mesenteric lymphatic malformation

## Abstract

Mesenteric lymphatic malformations (MLM) are rare, benign cystic lesions that arise from an abnormal development of lymphatic tissue. They can be complicated by obstruction, volvulus, and the rare occurrence of torsion. We report the case of a 33-year-old woman presenting with abdominal pain, initially suspected to be appendicitis. A contrast-enhanced computed tomography (CT) revealed a heterogeneous mass in the right lower abdominal quadrant over the jejunum mesentery with imaging signs of torsion, suggestive of a lymphatic malformation. Surgical resection and histopathological analysis confirmed the diagnosis. Although more frequent in pediatric patients, these entities can present in adults with varied manifestations. Imaging is crucial for diagnosing and guiding surgical resection, especially in complicated cases. This case highlights mesenteric lymphatic malformations in the differential diagnosis of abdominal pain and the need to assess for complications carefully.

## Introduction

Lymphatic malformations (LM) are benign lesions that arise from aberrant embryological development and sequestration of lymphatic tissue that fails to establish connections with the normal drainage pathways, forming isolated cystic spaces with considerable variability in content and size [1-3]. Up to 95% of LM are located in the head, neck, and axillary regions, while only about 5% occur in the mediastinum and abdomen. Within the abdominal cavity, the most frequent site of involvement is the small bowel mesentery (70% of intra-abdominal cases). Nevertheless, mesenteric lymphatic malformations (MLM) are rare, accounting for only about 1% of all LM [4-6].

MLM may be asymptomatic or cause diverse and challenging clinical presentations, including abdominal pain or nonspecific gastrointestinal symptoms. Complications include infection, bowel volvulus, obstruction, and the rare torsion. To the best of our knowledge, torsion of an MLM was specifically described only once by Addison et al. in 1953 [7]. Its clinical and imaging heterogeneity makes the diagnosis of a torsed MLM (tMLM) challenging [2,8,9]. We present a case of tMLM, which was prospectively suggested and confirmed surgically.

## Case presentation

A 33-year-old woman with a past surgical history of cesarean section and supernumerary kidney resection consulted the emergency department after three days of abdominal pain. The pain began in the epigastrium, progressively worsened, and migrated to the right flank, associated with nausea and vomiting. On examination, vital signs were normal, and the body mass index was 32 kg/m². The patient showed tenderness in the right flank and right iliac fossa with no peritoneal irritation signs. At admission, a negative pregnancy test, normal complete blood count, and unremarkable urinalysis (Table 1) led to the consideration of acute appendicitis.

**Table 1:** Laboratory data HPF: high-power field

Variable	Patient value	Reference range
Hemoglobin	12.3 g/dL	12-16 g/dL in women
Hematocrit	37.2%	36%-46% in women
Platelet count	227,000/µL	150,000-400,000/µL
White blood cells	6,500/µL	4,500-11,000/µL
Neutrophils	4,238/µL	1,800-7,500/µL
Urinalysis		
Color	Yellow	Yellow
pH	6	5-8
Protein	Negative	Negative
Glucose	Negative	Negative
Ketones	Negative	Negative
Bilirubin	Negative	Negative
Nitrites	Negative	Negative
Red blood cells	None	0-3 HPF
Epithelial cells	Few	Few
Bacteria	None	None

**Figure 1:**
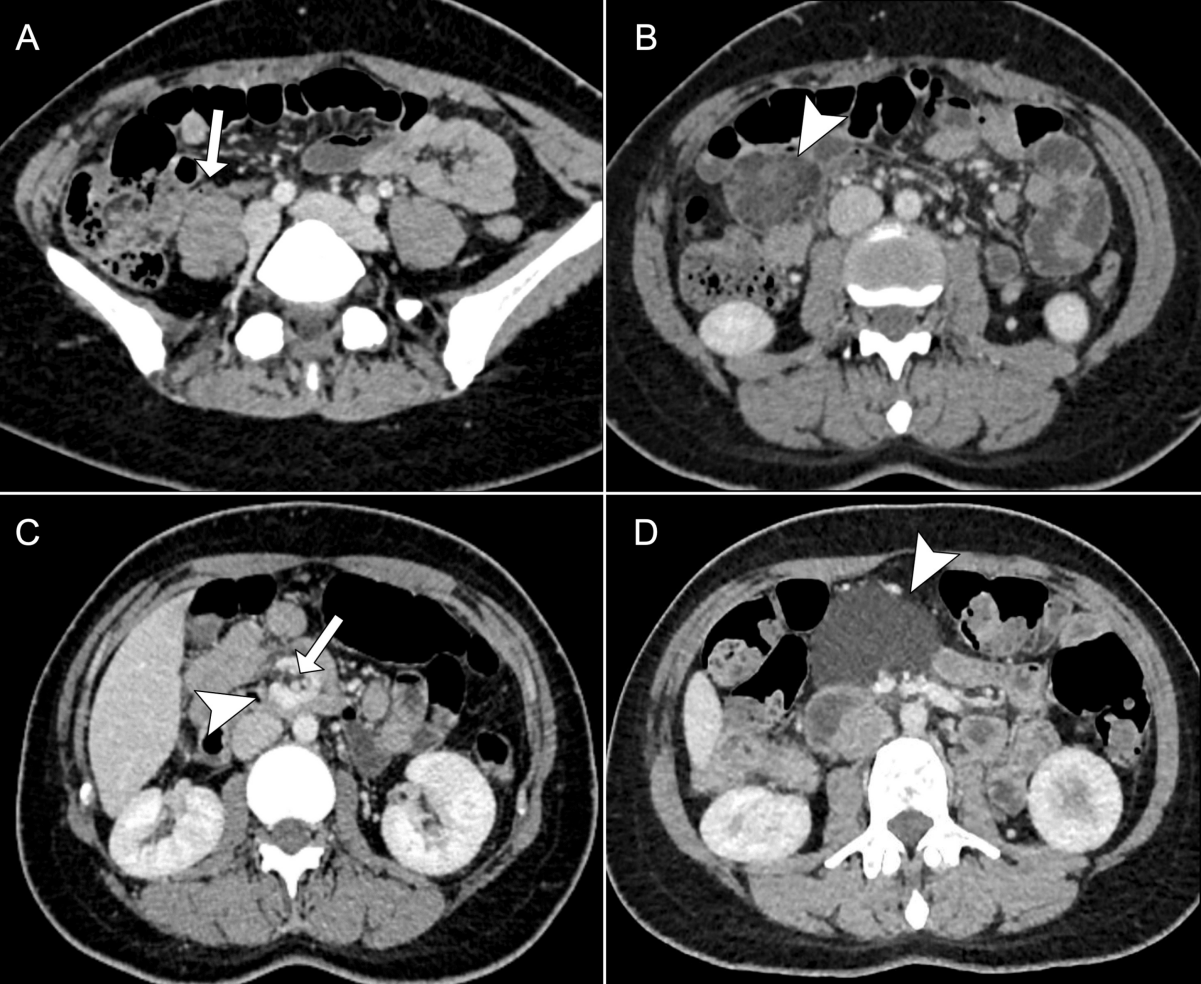
Current (A, B, and C) and previous (D) abdominal CECT in the portal venous phase, axial reconstructions, 1-mm slice thickness A: Axial image shows a normal cecal appendix (arrow). B: Mesenteric heterogeneous mass contacting jejunum in the right flank (arrowhead) with lobulated, well-defined margin, having fat and fluid densities (Hounsfield units range from -55 to +20), peripheral and internal septations with mild enhancement. There was no small bowel obstruction. C: Relationship between superior mesenteric artery (arrow) and superior mesenteric vein (arrowhead) with "whirl sign" suggesting torsion. D: Appearance of lesion on previous CECT (four months before), simple cystic mass with no noticeable walls (arrowhead), more centrally located and without the vascular "whirl sign" (not shown). CECT: contrast-enhanced computed tomography

Therefore, upon admission, an abdominal contrast-enhanced computed tomography (CECT) was requested, and appendicitis was ruled out (Figure 1A). However, it revealed a well-defined mass in the jejunum mesentery, with a lobulated margin, heterogeneous density including areas of fat attenuation, peripheral enhancement, striation of the adjacent mesentery (Figure 1B), and vascular "whirl sign" (Figure 1C). Shortly after the CECT acquisition, the surgery team looked for the body imaging division and discussed the differential diagnoses, including liposarcoma, low-flow vascular malformation, or cavitated mesenteric nodules. Upon further questioning, the patient reported several months of intermittent diffuse abdominal pain and an earlier abdominal CECT done four months before in another institution, which was required, accessed immediately (Figure 1D), and compared to the most recent one. Also, the previous laboratory workup was retrieved (Table 2).

**Table 2:** Three months previous laboratories AFP: alpha-fetoprotein, CA-125: cancer antigen 125, CEA: carcinoembryonic antigen

Variable	Patient value	Reference range
AFP	2 ng/mL	1.0-7.1 ng/mL
CA-125	23.2 U/mL	<35 U/mL
CEA	2.4 ng/mL	0-3.45 ng/mL

**Figure 2:**
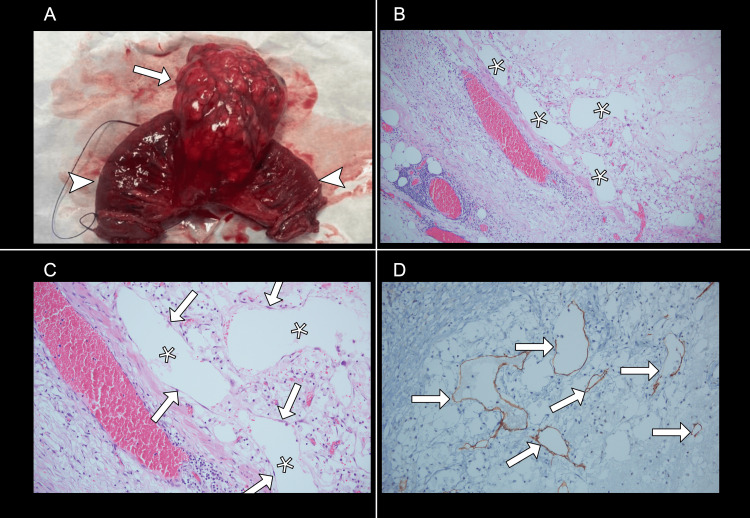
Jejunal-mesenteric lesion resection A: Surgical specimen with a lobulated mass measuring 8 × 5 × 4 cm (arrow) with nodular internal soft lesions involving the jejunum's mesenteric side (arrowheads). B and C: Hematoxylin and eosin staining at 100× and 200× magnifications, respectively, reveals numerous cystic spaces with proteinaceous content (asterisks) lined by flattened cells (arrows). There were also fibromuscular stroma and aggregates of lymphoid tissue. D: Immunohistochemistry with D2-40 at 200× magnification highlights membranous positivity in the lining cells of these vascular structures (arrows). D2-40 is a lymphatic endothelial marker, and its positivity supported the diagnosis of MLM. CD34 was also performed (not shown). MLM: mesenteric lymphatic malformations

The new mass density changes, shifted location, vascular whirl, and supportive negative tumoral markers were consistent with tMLM. After the clinical improvement of the patient's pain and a multidisciplinary consensus, the surgical team recommended surgical resection, which the patient accepted and was done on the second day of hospitalization. Laparoscopic surgery revealed a multiloculated, torsed mesenteric mass filled with clear and hemorrhagic liquid, firmly adhered to the jejunum, requiring jejunal resection and intestinal anastomosis. The histological analysis was consistent with lymphangioma and congestive changes (Figure 2). The patient recovered favorably and was later discharged. In a computed tomography six months after the surgery, there were no recurrence signs.

## Discussion

MLM, also known as mesenteric cystic lymphangiomas, mesenteric cysts, or omental cysts, are rare benign entities of lymphatic origin [2]. These lesions arise from congenital sequestered lymphatic tissue that fails to communicate with the normal lymphatic and venous systems at about 14-20 weeks of gestation [3,8-10]. Secondary causes include trauma, surgery, hemorrhage, inflammation, lymphatic obstruction, or radiation [1,11,12].

Over 80% of LM are diagnosed in the first year of life, with rare occurrence in adults, affecting men and women similarly. While the head and neck (75%) and axillary region (20%) are favored locations for LM, the remaining 5% occur in the mediastinum and abdomen. In the abdomen, the small bowel mesentery accounts for most cases (70%), nearly half involving the ileum [6]. Other less frequent abdominal locations are retroperitoneal, pancreatic, and splenic LM [1,6,12]. Specifically for the MLM, there is an estimated incidence ranging from 1/20.000 to 1/250.000 hospital admissions, with a male predominance (3:1) [6,11-13].

The clinical spectrum is broad. Patients with MLM can be asymptomatic or diagnosed as an incidental finding in most cases; others may experience abdominal pain, intermittent fever, abdominal distention, ascites, palpable mass, bowel or urinary obstruction, or acute abdomen mimicking surgical pathologies such as appendicitis, as our patient, pancreatitis, malignancies, or aortic aneurysms [2,8,11,14-16]. Other gastrointestinal symptoms, such as nausea, vomiting, and constipation, are also described [17]. Rare complications include bowel obstruction, volvulus, infarction, infection, bleeding, rupture, and torsion, which can worsen symptoms [1,9,11,15].

Radiologic findings are crucial for the detection and diagnosis of abdominal MLM. On ultrasound, MLM are multilocular cystic masses, anechoic or with echogenic debris, sometimes forming fluid levels [1,4]. On CT, the examination of choice, the lesions have circumscribed lobulated margins filled with hypodense fluid, sometimes with negative Hounsfield units if the cysts have chyle (chylangiomas or chylous cysts) and also with fluid layering [4,12,17]. Cyst wall and septa may be enhanced. Calcifications are an uncommon finding. In MRI, the mass shows a water signal in T1WI and T2WI, with possible hyperintense on T1WI due to hemorrhage or proteinaceous content, zones with decreased intensity on opposed-phase relative to the in-phase T1WI, fluid levels, or septal enhancement [18]. MRI is recommended in postmenopausal women with pelvic cystic masses to accurately differentiate MLM from gynecological cystic lesions [4,14].

When complications occur in MLM, the proteinaceous content changes the appearance of the cystic lesion and simulates a neoplastic mass, becoming symptomatic, denser due to hemorrhage, and associated with inflammatory changes [1]. In our case, the lesion position change with new internal denser zones, probably due to hemorrhage, and the new vascular whirl pointed toward tMLM.

The main differential diagnoses are fluid-containing masses in the abdomen, such as intra-abdominal abscesses, ovarian, pancreatic, enteric duplication, or mesenteric cysts, pseudocysts, ascites, urinoma, hematoma, lymphocele, epidermoid cysts, tuberculosis, hydatid disease, cystic mesothelioma, sarcomas, hemolymphangioma, and teratoma. Other intraperitoneal and hypodense lesions to consider in the neoplastic clinical scenario are lymph node metastasis or mucinous peritoneal implants, which, for the last scenario, tend to be multiple and with a known primary, typically appendiceal or ovarian mucinous neoplasm [1,2,8,9,19].

Percutaneous image-guided biopsy is not recommended due to the risk of dissemination of undiagnosed malignancy [8]. Although opinions are divided, when the finding is incidental or in asymptomatic patients, recommendations are shifting toward requiring only imaging follow-up to exclude rapid growth or the differential diagnoses [9,20]. Based on expert recommendation, follow-up could be made with clinical examination and abdominal ultrasound at 3, 6, and 12 months after treatment and annually thereafter [6].

When there are symptoms or complications, surgical resection may be indicated for management, requiring accurate imaging to identify anatomic location and distribution of the MLM, being important to achieve a complete excision and minimize recurrence risk [6,9]. The median size of the MLM at resection is 12 cm (range: 2-36 cm), and half of the patients taken to the operating room will also require bowel resection [11,13,14].

The prognosis is favorable but inversely proportional to lesion size and extension to other structures, with reported recurrence risks in 10%-27% even after an initial complete resection [6,9]. Although recent evidence suggests that when complete resection of cystic abdominal LM is achieved, there is no recurrence within a median follow-up of 12 months; however, more evidence and longer follow-up are needed [21]. A potential surgical complication is a chronic lymph fistula with uncontrollable ascites [15]. In non-surgical candidates, drainage or sclerotherapy (with ethanol, doxycycline, and bleomycin) may be considered, particularly for the macrocystic types, although with recurrence risks and perforation [6,9].

Histological analysis remains an essential step in confirming the diagnosis. It shows a circumscribed cystic lesion with or without endothelial lining, collagen, and fibrous tissue stroma, and focal aggregates of lymphoid tissue [2,3,8]. Immunohistochemistry markers that support the diagnosis of MLM are lymphatic vessel endothelial growth factor receptor-3, monoclonal antibody D2-40, and prox-1 [3,22].

Finally, this case report contributes to the very scarce and limited specific body of literature on tMLM, particularly by highlighting imaging signs that supported a radiologic presurgical diagnosis subsequently confirmed by surgery and pathology with no recurrence up to six months. Nevertheless, there are limitations, such as reference bias, since the discussion relies on published case reports and small case series. Furthermore, the rare presentation and extensive variability of LM limit the external validity and generalizability of our observations, including the imaging features described here. Also, the short-term follow-up limits knowledge about long-term outcomes and recurrence risk.

## Conclusions

We presented a case of a woman with an MLM complicated by torsion. MLM are congenital malformations of the lymphatic system and, although rare, should be considered in patients presenting with acute abdominal symptoms and radiologic findings of mesenteric cystic masses. When MLM becomes complicated, its typical appearance changes: hyperdense, inflammatory areas, vascular whirl, and displacement compared with earlier studies can help radiologists get back on the right track for diagnosis, guiding appropriate treatment. Published literature about tMLM remains limited to case reports or case series, emphasizing the importance of our paper and the need for further research.
